# Advances in Fingerprint Analysis for Standardization and Quality Control of Herbal Medicines

**DOI:** 10.3389/fphar.2022.853023

**Published:** 2022-06-02

**Authors:** Eka Noviana, Gunawan Indrayanto, Abdul Rohman

**Affiliations:** ^1^ Departement of Pharmaceutical Chemistry, Faculty of Pharmacy, Universitas Gadjah Mada, Yogyakarta, Indonesia; ^2^ Faculty of Pharmacy, Universitas Surabaya, Surabaya, Indonesia; ^3^ Center of Excellence, Institute for Halal Industry and Systems, Universitas Gadjah Mada, Yogyakarta, Indonesia

**Keywords:** herbal medicines, fingerprint analysis, chemometrics, chemical fingerprint, DNA fingerprint

## Abstract

Herbal drugs or herbal medicines (HMs) have a long-standing history as natural remedies for preventing and curing diseases. HMs have garnered greater interest during the past decades due to their broad, synergistic actions on the physiological systems and relatively lower incidence of adverse events, compared to synthetic drugs. However, assuring reproducible quality, efficacy, and safety from herbal drugs remains a challenging task. HMs typically consist of many constituents whose presence and quantity may vary among different sources of materials. Fingerprint analysis has emerged as a very useful technique to assess the quality of herbal drug materials and formulations for establishing standardized herbal products. Rather than using a single or two marker(s), fingerprinting techniques take great consideration of the complexity of herbal drugs by evaluating the whole chemical profile and extracting a common pattern to be set as a criterion for assessing the individual material or formulation. In this review, we described and assessed various fingerprinting techniques reported to date, which are applicable to the standardization and quality control of HMs. We also evaluated the application of multivariate data analysis or chemometrics in assisting the analysis of the complex datasets from the determination of HMs. To ensure that these methods yield reliable results, we reviewed the validation status of the methods and provided perspectives on those. Finally, we concluded by highlighting major accomplishments and presenting a gap analysis between the existing techniques and what is needed to continue moving forward.

## 1 Introduction

Herbal medicines (HMs) or herbal drugs have been known for centuries and empirically used to treat diseases by people across different cultures throughout history. Approximately 40% of the drugs used nowadays are directly or indirectly originated from natural products [plants 25%, microorganisms (13%), animals (3%)] (Calixto, 2019). While herbal medicines are generally perceived to be safer than synthetic drugs, the lack of regulations on HMs, especially in the past, has led to many adverse events (Walji et al., 2009). The contamination and adulteration of HMs also become a major concern (Posadzki et al., 2013). With the growing market of HMs, many countries have provided some regulations and guidance to ensure the safe use of the medicines by the patients or consumers. According to WHO, in 2018 about 90% of member states/countries have had national regulations on HMs (WHO, 2019). Many of these countries have 1) reported the use of national or other pharmacopeias (typically those from Britain, the United States, and Europe) or monographs that include HMs, 2) provided guidance for good manufacturing practices and mechanisms to ensure compliance with the manufacturing requirements, and 3) enforced special regulatory requirements for HMs or similar requirements to those for conventional pharmaceuticals. These regulations or policies are made and put into practice to create a standardized quality of herbal medicines and ensure their safety and efficacy.

The standardization and quality control of HMs involve the physicochemical evaluation of the raw/crude materials, assessment of the stability, efficacy, and safety of the finished products, and provision of product information to ensure the appropriate use by the consumers. To perform the evaluation, macroscopic and microscopic observations on the specimen as well as chemical and biological analyses are typically done. Analytical approaches to this evaluation generally fall into three categories: marker compound analysis or single component analysis (targeted analysis), fingerprinting/profiling, and metabolomic studies (Riedl et al., 2015). Marker compounds are commonly chosen from the abundant compounds in a botanical specimen and used as potency standards. However, the standardization of HMs based on marker compounds often yields unreliable results, especially when the chosen marker is not the biologically active component of the plant (Ruiz et al., 2016). In addition, the therapeutic effects of HMs may result from a complex interaction among various herbal constituents, and thus, a single or a few markers may not be a good predictor of the overall efficacy.

Fingerprinting techniques, on the other hand, interrogate the whole chemical profile of the botanical specimen. In combination with multivariate data analysis or chemometrics, this complex profile can be extracted into a common pattern that correlates with certain biological or pharmacological activities and set as a criterion for assessing the individual material or formulation (Kharbach et al., 2020). Tasks such as differentiating between authentic and adulterated HMs as well as distinguishing the origins of plant species have been performed using fingerprinting techniques (Wang and Yu, 2015; Sima et al., 2018). Chemical fingerprints are often generated using chromatographic techniques such as high-performance liquid chromatography (HPLC) and thin-layer chromatography (TLC) (Fan et al., 2006; Wagner et al., 2011; Custers et al., 2017). However, other techniques such as molecular spectroscopy, mass spectrometry, capillary electrophoresis, and DNA-based methods can also provide chemical or molecular fingerprints for such purposes. This review aimed to evaluate the progress and applications of these various fingerprinting techniques for the standardization and quality controls of herbal drugs.

## 2 Standardization of Herbal Drugs: Current Criteria, Limitations, and Challenges

Official methods for general standardization of herbal drugs or botanicals have been described by the current compendia and regulatory agencies (EMA, 2011; FDA, 2016; WHO, 2017; Farmakope Herbal Indonesia, 2017; Taiwan Herbal Pharmacopeia, 2019; British Pharmacopoeia, 2020; Hong Kong Chinese Materia Medica Standards, 2020; USP Herbal Medicine Compendium, 2021; USP44-NF39, 2021b, 2021c). Sampling methods for botanical samples have also been described (USP44-NF39, 2021b). The common standardization methods described by those official guidelines are macroscopic/microscopic characterizations, chemical tests, chromatographic fingerprinting, DNA profiling, quantitative determination of certain compounds/markers or a group of compounds, test for chemical contaminants/microorganisms, and physicochemical tests. According to WHO, non-specific chemical tests (e.g., phytochemical screening for alkaloids, flavonoids, terpenes, steroids, saponins, tannins, etc.) must not be applied for the identification (WHO, 2017). The general guidance for the development, validation, and standardization of new or non-official methods of analysis for botanicals/herbs and dietary supplements has been published by the Association of Official Analytical Chemists (AOAC, 2019). In this section, only the specific methods of herbal standardization/validation will be discussed in detail.

Some compendia and reference standards have described comprehensive macroscopic and microscopic characterizations of each of the herbs, including their pictures (Farmakope Herbal Indonesia, 2017; British Pharmacopoeia, 2020; Hong Kong Chinese Materia Medica Standards, 2020). However, due to phenotypic variations between different populations of identical species in commercial samples, these techniques may be insufficient for correct identification. To overcome this problem, the application of a DNA-based approach can be recommended for plant identification/authentication (Klein-Junior et al., 2021). It is worth noting that the DNA-based approach works well for plant authentication, but not for assessing the quality of plant materials. Many external factors can affect the (secondary) metabolites content of a plant both qualitatively and quantitatively. Plants with similar DNA profiles may not produce similar compositions of metabolites. Therefore, a quality control tool solely based on DNA profiling is not recommended. A combination of chemical characterization (both qualitative and quantitative) and DNA profiling/barcoding would serve as a more comprehensive method of herbs identification (Indrayanto, 2018a; Leong et al., 2020).

All the official guidelines described above generally apply high-performance thin-layer chromatography (HPTLC) for the method of identification of the herbs. Detailed HPTLC methods of identification for the article on botanical origins or herbs have been described (USP44-NF39, 2021a, 2021f). TLC identification of each herb, as described in USP, BP, Indonesian Herbal Pharmacopeia, Taiwan Pharmacopeia, USP Herbal Compendium, and Hong Kong Chinese Materia Medica Standards, is generally carried out by observation of the TLC plates under white light and UV light. For example, adulterants of *Sclorocarya birrea* leaves and leaf products could be visually detected on HPTLC images using white light and UV-366 nm (Do et al., 2020). Retardation factors (R_f_) of the target spots, their colors, and intensities can be compared between the sample and the standard. However, this visual observation may show poor reproducibility due to the strong influences of experimental conditions (Klein-Junior et al., 2021). To have more reliable results for the visual identification of botanicals using TLC profiles, a complete and comprehensive method validation should be performed (Reich et al., 2008; Do et al., 2021). The application of densitometry for the evaluation of TLC chromatograms is preferred due to its accuracy and precision. Method validation for the assessment of TLC fingerprinting using HPTLC-densitogram has been reported in which coefficient correlations (R), congruence coefficient (c), similarity index (SI), and dendrogram were applied for evaluating the densitograms of samples and standards (Srivastava et al., 2019). HPTLC-profile plots could also be directly generated using Image^R^ software after auto processing and enhancing the TLC images by XnView 2.40^R^ freeware, followed by chemometrics evaluation (Ibrahim and Zaatout, 2019). To obtain a more accurate and reproducible assessment of the TLC profile, the application of similarity analysis as described in the Chinese Pharmacopoeia 2015 is recommended (Shen et al., 2021). The applications of HPLC fingerprints for the authentication of Chinese herbs have also been described by Hongkong Chinese Materia Medica Standards. Typical HPLC chromatograms and relative retention time (RR_t_) of specific compounds are provided in each monograph (Hong Kong Chinese Materia Medica Standards, 2020).

Various analytical techniques can be applied for generating chemical fingerprinting of HMs. These include spectrometric methods (e.g., infrared (IR), near-infrared (NIR), nuclear magnetic resonance (NMR), and mass spectrometry (MS)), chromatographic methods (e.g., HPLC, gas chromatography (GC), HPTLC), and capillary electromigration methods (e.g, capillary electrophoresis (CE)) combined with various detectors. The quality of the fingerprints depends on sample pre-treatment and the selected chemical techniques (Klein-Junior et al., 2021). Typically, the more sophisticated the instruments, the more reliable the results. However, the operational cost for these sophisticated methods could be expensive. These methods also need highly trained personnel and large amounts of reagents and solvents. It is well known that all methods used at a QC laboratory should not only be simple, cost-effective, and fast, but they also need to be accurate and precise. Therefore, direct attenuated total reflectance-Fourier transform infrared (ATR-FTIR) spectroscopy combined with chemometrics could be recommended as an alternative QC tool to the HPTLC (i.e., the commonly used method in pharmacopeias). The ATR-FTIR requires minimum to no sample preparations. To apply FTIR methods, the availability of official botanical reference materials (BRMs) is crucial (Indrayanto, 2018a). If the BRM is not yet available, the botanical or herb reference materials must be standardized first using liquid chromatography-high resolution tandem mass spectrometry (LC-HR-MS/MS) to evaluate their exact biochemical components.

All official guidelines describe the method of quantification of a certain compound(s) or a group of compounds using spectroscopic and/or chromatographic methods. Minimum concentrations of the compounds are generally described and used as the acceptance criteria. However, these criteria are not appropriate to be applied as a parameter of the quality of herbs (Länger et al., 2018). The assayed compounds/markers must be considered as purely analytical markers without correlation to quality or efficacy. Chemical constituents (compounds) in botanical articles can be categorized as active principles, active markers, analytical markers, or negative markers (USP44-NF39, 2021c). Unfortunately, in all monographs of botanicals in the current USP 44-NF 39, those terms of markers are not described. Only the name of the compounds or a group of compounds and their minimum concentrations or specification range concentrations (for certain preparations) are described by each monograph. Analyzing a certain compound or a group of compounds in herbs will only be useful for QC if the compounds have a direct correlation to the efficacy or toxicity of the herbs. For this purpose, some researchers have recommended new criteria for the QC markers e.g. Herb MarRS system, Q-marker, Bioactive chemical markers, etc., or in general terms referred to as “quality markers” (Indrayanto, 2018a). If an herb is already known for its quality markers, there is no need to evaluate the quality of the herb using the fingerprinting method.

Most commercial herbal drug preparations consist of a mixture of herbs and/or extracts. Therefore, methods for standardization of individual herbs that are described in the Pharmacopeia cannot be applied directly as a quality control (QC) tool for all stages of the manufacturing processes i.e. incoming materials, in-process control, finished product, and sample storage. To obtain a consistent quality of HMs, all stages of the production line need to be evaluated by QC (Indrayanto, 2018a). If the quality marker of each component of the assessed herbal product is known, the QC can be performed by analyzing those markers in all stages of production. However, if the quality markers are not yet known, the QC should be performed by using a combination of fingerprinting methods and chemometrics, both qualitatively and quantitatively. Each batch of a commercial herbal drug should show identical efficacy. Thus, the exact composition of herbs and/or extracts should be determined (Heinrich et al., 2020). For this purpose, the combination of chemical profiling and chemometrics seems to be the method of choice. Generally, a combination of various analytical techniques needs to be applied to the analysis of HM preparations (Muyumba et al., 2021). The authors recommend starting with a sophisticated method to define the exact composition of the HM (e.g., LC-HR-MS/MS or NMR 400 MHz), and then transferring the method to a relatively cost-effective method (e.g., ATR-FTIR, HPTLC, LC-UV/Vis) for routine QC assessment. Procedures for the method transfer have been described in compendia (USP44-NF39, 2021d).

## 3 Fingerprint Data Analysis Using Chemometric Approach

Quality assessment of HMs using a combination of fingerprinting and chemometrics is the method of choice if the quality markers are not yet specified. Chemical profiles/fingerprints of HMs obtained by instrumental methods such as LC-MS/MS and ^1^H-NMR spectroscopy feature similarities and differences among one another. These sets of similarities and differences enable classifications of samples into certain categories, for instance, authentic and adulterated HMs. However, in any HMs, there are a large number of chemical responses from unknown components, which make the data handling challenging. And hence, powerful statistical techniques known as chemometrics are typically employed to treat these large chemical data (Nikam et al., 2012). The combination of chemical fingerprints and chemometrics enables accurate identification of samples even if the samples do not contain chemically characteristic constituents at exactly similar concentrations.

**FIGURE 1:**
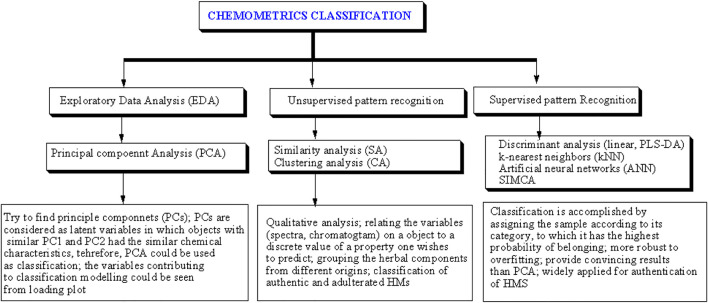
The chemometrics classification techniques widely applied for the identification of objects. Reproduced from (Rohman and Windarsih, 2020) under the terms and conditions of the Creative Commons Attribution (CC BY) license. PLS-DA = partial least squares discriminant analysis, SIMCA = soft independent modeling of class analogy.

Chemometrics can be defined as the utilization of statistics and mathematics to analyze chemical data (Rohman and Putri, 2019). The International Chemometrics Society defined chemometrics as “*the science of relating chemical measurements made on a chemical system to the property of interest (such as concentration) through the application of mathematical or statistical methods*” (Huang et al., 2015). Chemometrics is widely applied to chemical data obtained from measurements using spectroscopic and chromatographic methods (Rohman and Windarsih, 2020). Chemometrics of classification is the most common chemometrics technique applied in fingerprint profiling of HMs. The aim of classification chemometrics is to correlate the chemical data or variables obtained from instrumental measurements to a discrete value of a property the analyst wishes to predict (Biancolillo and Marini, 2018). These techniques include exploratory data analysis and pattern recognition (either supervised or unsupervised) (Figure 1). Exploratory data analysis and unsupervised pattern recognition can be applied to reduce the amount of original data and gain a better understanding of the chemical data sets (Berrueta et al., 2007). Multivariate calibrations can be used for building the prediction models for the analyte(s) of interest. The most popular multivariate calibrations applied in standardization and quality controls of HMs included principal component regression, partial least squares regression (PLSR), and multiple linear regression (MLR) (Singh et al., 2013).

Principal component analysis (PCA) is widely used for exploratory data analysis. PCA is capable of reducing the dimensionality of the datasets and increasing the interpretability of the data while retaining the most important piece of information of the datasets (Jollife and Cadima, 2016). This task is achieved by generating principal components (PCs) as an orthogonal linear transformation that considers variances from different variables in the data (Rawat et al., 2021). Unsupervised pattern recognition algorithms such as similarity analysis (SA) and hierarchical clustering analysis (HCA) are also commonly applied. SA can be applied to classify HMs samples based on the correlation and congruence coefficients. If the correlation and congruence coefficients are close to 1, it can be stated that the two fingerprints are similar (Chuchote and Somwong, 2019). HCA, on the other hand, can be performed to reveal the highest similarity within a cluster and the highest dissimilarity among clusters (Chuchote and Somwong, 2019).

Supervised pattern recognition algorithms are typically used to generate classification models and determine which class a sample belongs to. Classification models are built based on data obtained from chemical measurements of known samples. Unknown samples are then assigned to a previously determined class based on the similarity of chemical properties between the sample and the known class members. Supervised pattern recognition algorithms such as linear discriminant analysis (LDA), partial least squares discriminant analysis (PLS-DA), soft independent modeling of class analogy (SIMCA), artificial neural networks (ANN), k-nearest neighbors (KNN), and least squares-support vector machine (LS-SVM) are often applied in HM authentication (Huang et al., 2015). LDA can maximize the distance between classified samples or groups, enabling better differentiation among HM classes (Luo and Shao, 2013). In the case of the number of samples is less than the number of measured variables (often referred to as ‘ill-conditioned), PLS-DA can be applied to improve the capability of the predictive models to classify the samples (Razmovski-Naumovski et al., 2010). By applying PCA to each class, SIMCA classification can be generated to produce predictive models using the significant components only (Qu and Hu, 2011). KNN algorithm places samples and their relative distances among one another based on the level of similarity (Harrison). Similar things exist in proximity and *vice versa*. Another supervised method, LS-SVM which is based on the theory of statistical learning using classification and regression techniques, can also be employed in HM fingerprint profiling (Samui and Kothari, 2011).

### 3.1 Validation of Chemometrics Techniques

Chemometrics is mainly based on the application of empirical models intended for building predictive models either for qualitative (classification) or quantitative (calibration) purposes. The experimental measurements could provide large data containing a lot of information which allows the analyst to make the predictions of one or more properties of interest. The selection of an appropriate chemometrics model and the way to verify the model reliability are fundamental aspects to consider. The chemometrics strategies for performing these tasks are collectively referred to as validation. Validation aims to evaluate whether reliable conclusions can be drawn from the chemometrics modeling (Brereton et al., 2018). During the validation process, it is suggested to include some criteria such as the appropriateness of the chemometrics model, the adequacy of computational calculations used in the fitting procedure, the statistical reliability of models, and the generalizability of any resulting interpretations (Westad and Marini, 2015).

To assess the performance of the developed models, some diagnostic parameters based on the model or the calculation of residuals (i.e., differences between actual and predictive parameters) are often used as error criteria. Validation of chemometrics models can be performed using two approaches, internal validation (cross-validation) and external validation (Miller and Miller, 2018). Cross-validation is required to avoid overfitting the model. Cross-validation is based on the repeated resampling of the dataset into the sub-sets of training and testing. In multivariate calibration models, this validation is typically done using the leave-one-out technique. In this technique, one of the calibration samples is taken out and the remaining calibration samples are used to establish a new calibration model. The removed sample is then evaluated using the newly established model. This process is repeated to evaluate each calibration sample. Cross-validation can be chosen if the number of the evaluated samples is small and it is not feasible to build an external test set. The main disadvantage of cross-validation is that the resulting estimates may still be biased because the calibration and validation datasets are never completely independent. External validation employed two separate data sets for the calibration and validation. In this approach, the residuals are calculated from independent samples which mimic how the developed model will be routinely used. Therefore, this strategy is recommended whenever possible (Biancolillo and Marini, 2018). The validation approach should be selected based on the sample size (Kos et al., 2003). When the dataset or number of samples is small (less than 50), cross-validation is preferred, while external validation should be used if the number of samples is more than 50.

To evaluate the classification chemometrics, some performance characteristics including sensitivity, specificity, precision, accuracy and model efficiency are used (Oliveri and Downey, 2012; Oliveri et al., 2020):

$$\text{Sensitivity}=\;\frac{\text{TP}}{\text{TP}+\text{FN}}$$ (1)

$$\text{Specificity}=\;\frac{\text{TN}}{\text{TN}+\text{FP}}$$ (2)

$$\text{Efficiency}=\sqrt{\frac{\text{TP × TN}}{\left(\text{TP}+\text{FN}\right)\text{ × }\left(\text{TN}+\text{FP}\right)}}$$ (3)

$$\text{Precision}=\;\frac{\text{TP}}{\text{TP}+\text{FP}}$$ (4)

$$\text{Accuracy}=\;\frac{\text{TN}+\text{TP}}{\text{TN}+\text{TP}+\text{FN}+\text{ FP}}$$ (5)

where TN is true negative, TP is true positive, FN is false negative, and FP is false positive. A parameter known as Matthews’s correlation coefficient (r_M_) can also be used as a comprehensive evaluation of model efficiency which considers all four possible outcomes (i.e. TP, TN, FP, and FN).

$${\text{r}}_{\text{M}}=\;\frac{\left(\text{TP × TN}\right)-\left(\text{FP × FN}\right)}{\sqrt{\left(\text{TP}+\text{FP}\right)\text{ × }\left(\text{TP}+\text{FN}\right)\text{× }\left(\text{TN}+\text{FP}\right)\text{× }\left(\text{TN}+\text{FN}\right)\;}}$$ (6)

Some statistical parameters typically used for the performance characteristics are coefficient of determination (R^2^) and root mean square error (RMSE). R^2^ determines the relationship between two variables: actual values and predicted values from the instrument (accuracy). The precision of the validated analytical method is assessed by root mean square error of calibration (RMSEC) for error evaluation in the calibration model and root mean square error of prediction (RMSEP) for error evaluation in the prediction model. RMSEC and RMSEP can be obtained using the following equations:

$$\text{RMSEC}=\sqrt{\frac{\sum_{\text{i}=1}^{\text{m}}{\left(\text{Ŷ}\text{i}-\text{Yi}\right)}^{2}}{\text{M}-1}}$$ (7)

$$\text{RMSEP}=\;\sqrt{\frac{\sum_{\text{i}=1}^{\text{n}}{\left(\text{Ŷ}\text{i}-\text{Yi}\right)}^{2}}{\text{N}}}$$ (8)

M and N are the numbers of samples used in calibration and validation, respectively. Yi and Δi are the predicted and actual values, respectively. Root mean square error of cross-validation (RMSECV) is used to express RMSEC if cross-validation using the leave-one-out technique is used.

For qualitative identification of HM, validation samples should consist of specified inferior test material (SITM) and specified superior test material (SSTM) (AOAC, 2019). SITM is a botanical (herb) mixture that has the maximum concentration of target material (herb) that is considered unacceptable (negative result) as specified by its standard method performance requirements (SMPR). SSTM is a mixture of herbs that has the minimum concentration of the target herb that is considered acceptable (positive result). The detailed procedure can be found in the AOAC guidelines (AOAC, 2019). BRM of each specified herb should be used for preparing SITM and SSTM. The availability of authentic herb/botanical reference materials and/or stable standardized mixtures of herbs and/or extracts with exact compositions as validation samples are crucial for qualitative and quantitative assessment of HMs using chemical profiling or fingerprinting (Wulandari et al., 2022).

General official validation methods for chemometrics analysis have been described in USP 44-NF39 general chapter (1039) Chemometrics (USP44-NF39, 2021e) and EP 10.0, 5.21 Chemometrics methods applied to analytical data (European Pharmacopoeia, 2021). Quantitative methods of analysis using chemometrics should be validated in two steps. First, calibration models are evaluated based on the R^2^, RMSEC or RMSECV, and RMSEP (European Pharmacopoeia, 2021; USP44-NF39, 2021e). If the model meets the requirements of the analytical target profile, then the validation can be continued to step 2, which is the evaluation of general validation parameters of drugs (i.e., accuracy, precision, and robustness). This evaluation should be performed according to the general chapters of the USP 44-NF 39 (1225) (USP44-NF39, 2021 h), (1210) (USP44-NF39, 2021 g), and/or the AOAC guidelines (AOAC, 2019). Unfortunately, many published works do not report the method validation completely, as described in Tables 6, 7, and 8 of our recent book chapter (Wulandari et al., 2022). Without method validation, the reliability of the reported data cannot be ascertained. Detailed discussions regarding the development, validation, and standardization of analytical methods (qualitative and quantitative) using chemometrics have been previously described (Rohman and Windarsih, 2020; Wulandari et al., 2022).

## 4 Applications of Fingerprinting Methods

Due to their separation capability, FDA and the European Medicines Agency recommend chromatographic techniques for the standardization of HMs. Consequently, many chromatographic methods have been developed for fingerprinting/profiling different HMs samples in the past decades. Among these, TLC, HPTLC, HPLC, LC-MS/MS, capillary electrophoresis, and GC equipped with several detectors were used (Sima et al., 2018). Vibrational spectroscopy and NMR spectroscopy combined with chemometrics have also emerged as analytical tools for the standardization of HMs. Vibrational spectroscopy (VS) methods including FTIR, NIR, and Raman spectroscopy generate spectra containing useful information for the standardization of HMs and have been widely used (Moros et al., 2010). VS methods are rapid and the generated fingerprint spectra can be treated using chemometrics to yield more interpretable results for answering different biological questions (Wang and Yu, 2015; Rohaeti et al., 2021). NMR spectroscopy has also emerged as a powerful analytical technique for the QC of HMs because it can be used for simultaneous and rapid analysis of primary or secondary metabolites with good sensitivity (Kim et al., 2010; Imai et al., 2020). Chemical fingerprints can also be obtained from MS spectra which readily provide information on the presence of certain metabolites or elements within the HM samples based on their masses (Yang and Deng, 2016). Besides chemical fingerprints, molecular fingerprints from the plant DNA can serve as a great authentication and standardization tool (Zhokhova et al., 2019). Some advantages and disadvantages of these different methods in combination with chemometrics are shown in Table 1.

**TABLE 1:** Advantages and disadvantages of fingerprinting methods for the standardization and quality control of herbal medicines.

Methods	Advantages	Disadvantages	References
Vibrational spectroscopy (NIR; mid-IR and Raman)	Rapid, applicable to both raw materials and processed samples (e.g,. extracts, finished HM products), requires minimum to no chemical solvents and reagents during sample pretreatment, non-destructive, enables online analysis	No separation capacity, standardization and quality control of HMs with complex components must be supported by chemometrics methods	Li et al. (2020c), Huck (2015)
NMR spectroscopy	Non-destructive and non-invasive, environmentally friendly, relatively rapid and easy to use on a regular basis, minimum sample preparation, can provide structural information of components of complex mixtures without pre-isolation/purification, suitable for metabolite fingerprinting of HMs	Low sensitivity, signal overlapping in complex HMs, relatively sophisticated and expensive instruments, the use of chemometrics software is inevitable to treat the large data generated	Pacholczyk-Sienicka et al. (2021)
Chromatography	Can separate target compounds in HM matrices into fractions or isolated compounds, wide suitability, high resolution, selectivity, sensitivity, and can be fully automatable operation; By using HR-MS/MS detector, the chemical structure of target peaks can be predicted and determined	Time-consuming, needs extraction and stability studies for standards and samples, high cost for sophisticated instruments (LC-MS/MS); Needs peak alignments and retention time correction for each of samples prior to multivariate analysis	Kharbach et al. (2020), Li et al. (2020b)
Capillary electrophoresis	High separation capability, can be applied in either single marker analysis, fingerprinting, or metabolomic studies for quality control of HMs, low sample and reagent consumption, relatively lower cost of instrumentation compared to HPLC/GC.	Low resolution for nonpolar or noncharged analytes unless coupled with other partition-based separation techniques, lower sensitivity compared to chromatographic techniques due to the low amount of sample used	Hou et al. (2019), Gong et al. (2021)
Direct MS	Capable of rapidly separating analytes in complex HM matrices based on the mass per charge, many ambient MS techniques are available and require only minimal to no sample preparation, some techniques support the imaging of chemical fingerprints	MS detector is relatively more expensive than other detectors, ionization efficiency may vary among techniques and/or sample matrices which could result in low reproducibility	Freitas et al. (2019), Pereira et al. (2021), Zhang et al. (2022)
DNA barcoding/fingerprinting	Enable authentication of medicinal samples to species level, can detect adulteration from even closely related species, suitable for plant genotyping to create standardized medicinal crops	Does not provide any information on metabolite contents of the HMs, cannot detect adulterants from different parts of plants from the same species	Hadipour et al. (2020), Hadipour et al. (2020)

### 4.1 Spectroscopic Fingerprinting

FTIR and NMR spectroscopies are commonly used for QC and herbal authentication through fingerprint profiling. FTIR is one of the most commonly used spectroscopic techniques, which can detect microgram levels of samples (Arendse et al., 2021). FTIR spectroscopy operates in the mid-IR region which corresponds to wavenumbers of 4,000–400 cm^−1^. Functional groups of HM components can absorb IR photons at specific frequencies (or wavenumbers), resulting in fingerprint spectra that can be used for herbal authentication (Azlah et al., 2020). To assist data interpretation, chemometrics tools, mainly exploratory data analysis and pattern recognition, can be used.

The QC of different extracts of *Sonchus arvensis* (known locally in Indonesia as *Tempuyung*) was successfully carried out by FTIR spectroscopy combined with chemometrics (Rafi et al., 2021). PCA was used for the classification of the extracts, while PLS was applied for finding functional groups responsible for the antioxidant activity. FTIR spectra at combined wavenumbers of 3,200–2,800 cm^−1^ and 1800–400 cm^−1^ that were previously subjected to pre-processing using standard normal variate provided distinct clusters for the different extracts. PC1 and PC2 described 95% of the total variance within the dataset (PC1 and PC2 explained 79% and 16% of the variance, respectively). According to the loading plot of the PLS regression, the O–H (at 3,500–3,300 cm^−1^) and C–O (at 1,205–1,124 cm^−1^) bonds, which are attributed to the phenolic compounds, gave a significant contribution to the antioxidant activity of *S. arvensis* leaf extracts. A similar approach was also used for the QC of *Phyllanthus niruri* plants that were grown at different altitudes (Kartini et al., 2021).

ATR-FTIR and Raman spectroscopy have been used for the QC of turmeric that was adulterated with metanil yellow (MY), a toxic azo dye (Dhakal et al., 2016). Due to its similar appearance to turmeric, MY may be added to turmeric powder. FTIR spectra at 650–4,000 cm^−1^ can be used to detect the presence of MY in turmeric. Peaks between 1,628–1740 cm^−1^ were specific to turmeric. These peaks are correlated to the vibration of carbonyl groups, which are absent in MY. Moreover, the peak at 1,140 cm^−1^ was specific to MY and demonstrated a linear correlation between the actual and predicted concentrations of MY (R = 0.95). Raman spectroscopy at 100–3,700 cm^−1^ could also be used for the quantification of the adulterant. A Raman peak at 1,406 cm^−1^ showed a linear correlation (R = 0.93) between the actual and predicted concentrations of MY. FTIR could detect MY at 5% concentration, whereas Raman could detect down to 1%. However, the performance characteristics including accuracy and precision were not stated. Both parameters are required to assess systematic and random errors, respectively.

Proton (^1^H) and carbon (^13^C) NMR spectroscopy are extensively applied for the QC of HMs due to the unique fingerprints generated from the interaction between molecules and certain radio waves. This interaction results in changes in the spin direction. With the development of two-dimensional (2D) NMR techniques such as J-resolved, heteronuclear single quantum correlation, and heteronuclear multiple bonds correlation, the techniques have the potential to be standardized as analytical fingerprinting techniques for HM standardization (Sun et al., 2018).

**FIGURE 2:**
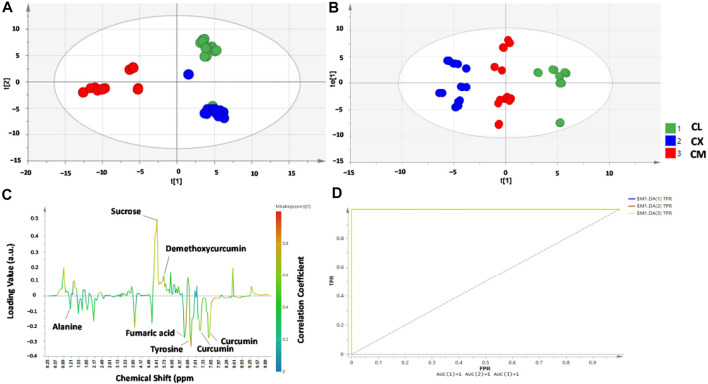
Partial least square-discriminant analysis (PLS-DA) score plot **(A)**, orthogonal projections to latent structures-discriminant analysis (OPLS-DA) score plot **(B)**, OPLS-DA S-line correlation plot **(C)**, and receiver operating characteristic (ROC) curve **(D)** for differentiation and classification of *C. longa* (CL)*, C. xanthorrhiza* (CX)*, and C. manga* (CM) from different origins. Reproduced from Nuraini et al. (2021) under the terms of Creative Commons Attribution (CC BY) license (https://creativecommons.org/licenses/by/4.0/).

NMR combined with chemometrics have been applied to classify HM samples based on their geographical origins. Recently, the suitability of ^1^H-NMR coupled with PCA and orthogonal PLS-DA (OPLS-DA) was reported for the differentiation of three *Curcuma* species namely *C. longa, C. xanthorrhiza*, and *C. manga* from different origins in Indonesia (Nurani et al., 2021). There are 14 metabolites identified from the ^1^H-NMR spectra that are responsible for generating the classification model. These metabolites include curcuminoids (curcumin, dimethoxy- and bis-desmethoxycurcumin), some carbohydrates, and amino acids. In addition, NMR in combination with PCA and OPLS-DA could differentiate *C. longa, C. xanthorrhiza, and C. manga* from different origins as shown in Figure 2. The validation results, as carried out using the permutation test, indicate that the developed model demonstrated goodness of fit (R^2^ value >0.8) and good predictivity (Q^2^ >0.45).

**FIGURE 3:**
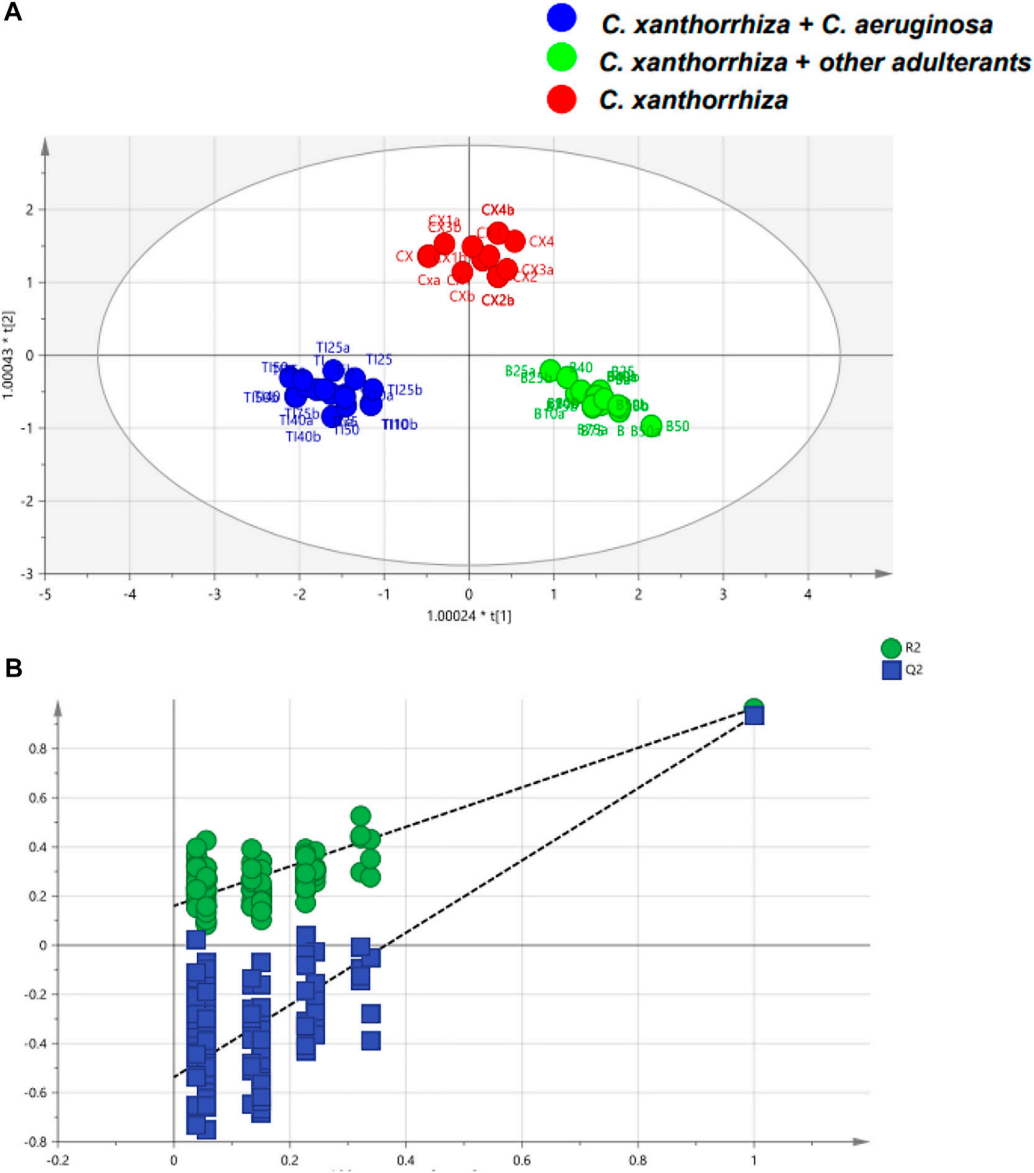
The orthogonal projections to latent structures-discriminant analysis (OPLS-DA) score plot of pure and adulterated *C. xanthorrhiza* with *C. aeruginosa* **(A)** and permutation test of OPLS-DA model **(B)**. Reproduced from Rohman et al. (2020) under the terms of Creative Commons Attribution (CC BY) license (https://creativecommons.org/licenses/by/4.0/).

Another geographical classification was reported for Asian red pepper powders that were distributed in Korea (Lee et al., 2020). Analysis of the ^1^H-NMR spectra showed that several metabolites played significant roles in differentiating the samples. For example, higher tyrosine and alanine contents were found in samples from Vietnam, whereas the quantities of *a*-glucose, *ß*-glucose, adenosine, and tryptophan were higher in samples from Korea. Using canonical DA, 15 blind samples were correctly classified and one sample from China was misclassified due to the high contents of *a*-glucose and *ß*-glucose. Difference in sugar contents between HMs was also reported from serrano pepper grown in two areas in Mexico: Veracruz and Oaxaca (Becerra-Martínez et al., 2017). There was a distinct difference in the concentrations of metabolites including glucose, fructose, sucrose, and citrate between the two sample groups. In addition, lactate was only present in samples from Oaxaca whilst succinate was only detected in Veracruz samples. Differentiation using PCA provided R^2^ and Q^2^ values of 0.936 and 0.875, respectively. A better classification was obtained with OPLS-DA with R^2^X = 0.923, R^2^Y = 0.999, and Q^2^ = 0.996. NMR in combination with PCA and OPLS-DA is also successful for the classification and authentication of *C. xanthorrhiza* from adulterant of *C. aeruginosa*. The decreased contents of curcumin as determined by HPTLC in adulterant levels of ≥40% of *C. aeruginosa* in *C. xanthorrhiza* rhizome could indicate the adulteration practice of *C. xanthorrhiza* with other rhizomes. Morever, OPLS-DA is successfully applied for the classification of pure and adulterated *C. xanthorrhiza* with higher R2X (0.965), R2Y (0.958), and Q2 (cum) (0.93) as shown in Figure 3 (Rohman et al., 2020).

Using an appropriate selection of variables obtained from molecular spectroscopic measurements (NIR, FTIR, NMR) and suitable chemometrics techniques, quality and standardized HMs can be obtained. In addition, the combination of chemometrics and spectral datasets is a proven tool to authenticate HMs from adulterants and assure the geographical origins of the HMs. Authentication by spectroscopic methods must be accompanied by the analysis of certified reference herbal materials or product formulations containing similar compositions to those of raw materials or products being investigated (Coskun et al., 2021).

### 4.2 Chromatographic Fingerprinting

Due to the complexity of plant materials and their extracts, chromatographic methods (e.g., TLC, HPLC, and GC) coupled with spectroscopic detectors (UV/diode array detector (DAD), MS, NMR) are mostly applied as standardization methods of herbal drugs. Since different compounds could have identical UV spectra, MS or HR-MS/MS becomes the detector of choice. While LC-NMR can also be applied, the cost of analysis using this instrument could be expensive. Some recent reviews (2020–2021) discussing the application of a combination of chromatographic fingerprinting and chemometrics for the quality assessment of herbal drugs have been published (Balekundri and Mannur, 2020; Li et al., 2020b; Kharbach et al., 2020; Shen et al., 2020; Klein-Junior et al., 2021). Generally, the objectives of fingerprinting methods are 1) to determine seasonal and geographical locations, 2) taxonomic identification, 3) assessment of extraction processes, and 4) quality control and authentication of the herbal drugs (Kharbach et al., 2020). If the chromatographic fingerprint of an herbal drug is identical to its botanical reference material or standardized extract, it can be assumed that the herb shows a phyto equivalence to the standard (Sahoo et al., 2010). Phyto equivalence means the bioactivities and/or toxicities of the herbal drug are identical to its standard based on the similarity of their metabolite contents. Therefore, chromatographic fingerprinting can be applied as a standardization tool for ensuring the efficacy and safety of herbal drugs if their quality markers have not been yet specified.

Chromatographic fingerprinting can be categorized into characteristic chromatograms and fingerprints. A characteristic chromatogram is a chromatogram that allows the selection of one or several components/peaks as an identification marker for the quality control of HMs. Fingerprint analysis is a semi-quantitative analysis based on the whole peaks or components in the samples. Both methods are described in the Chinese Pharmacopoeia 2015 (Shen et al., 2020). Most of the current Pharmacopoeias, as described in Section 2, applied HPTLC fingerprints for the identification of herbs. The accuracy of the fingerprint as a standardization tool depends on the instrument, the more sophisticated the instrument the more accurate the fingerprints are. To our knowledge, LC-HR-MS/MS is the most accurate method for performing chromatographic fingerprinting. GC-MS is the method of choice for thermo-stable samples. For complex samples, pre-treatments using headspace solid-phase microextraction (HS-SPME) can be applied (Balekundri and Mannur, 2020).

The drawbacks of chromatography are time-consuming sample preparation and/or extraction prior to chromatographic measurements (Li et al., 2020b). The extraction method could affect the metabolite profile of the samples. Thus, the selection of extraction solvents is crucial. The stability of the metabolites in the selected solvents must also be evaluated. The stability of metabolites can be evaluated by calculating the similarity values (i.e., R and c must be close to 1), performing PCA on the chromatograms, and observing variation/relative standard deviation (RSD) in the retention time of 5–10 important peaks of the QC samples (tight clustering) that are stored for a certain time. For example, the metabolites are considered stable if the RSD of the R_t_ is less than 5% (Indrayanto, 2018b). Bingbing et al. recently studied the stability of herbal extracts at 0, 4, 6, 8, 12, and 24 h time intervals (Bingbing et al., 2021). Six peaks in the GC-MS total ion chromatogram were selected as markers for determining the retention time (R_t_) and peak area (PA). RSDs of the R_t_ and peak area from the 6 peaks were <0.04% and <10%, respectively. For the validation of chromatographic fingerprinting methods, precision (intra- and inter-day) should be then evaluated using QC samples if the stability of QC samples can be confirmed (Indrayanto, 2018b).

The raw data of the first order chromatographic fingerprinting (R_t_/R_f_ vs intensity of detector response) should undergo pre-processing prior to further evaluation using chemometrics. Some of these pretreatments include baseline correction, smoothing, and peak alignment. Numerous approaches for peak alignment and retention-time correction, including correlation optimized warping, parametric time warping, target peak alignment, dynamic time warping, fuzzy warping, and semi-parametric time warping, have been generally applied (Li et al., 2020b; Kharbach et al., 2020). Examples of chromatographic methods applicable for the first-order chromatographic fingerprinting are HPLC-fixed UV/Vis, HPLC-evaporative light scattering detector (ELSD), HPLC-refractive index detector (RID), GC-flame ionization detector (FID), HPTLC-densitometry. However, the identity and purity of the observed peaks cannot be confirmed absolutely by the first-order fingerprint method. Peaks that have identical R_t_ or R_f_ do not necessarily represent identical compounds, and a single peak may contain more than one compound.

Second-order chromatographic fingerprints can be obtained using HPLC-DAD, LC-MS, and GC-MS. The data consist of pair data of R_t_ or mass-per-charge (m/z) or wavelength vs intensity. Because many structurally related compounds have similar UV/Vis spectra, the applications of HPLC-DAD fingerprinting have limitations (Klein-Junior et al., 2021), leaving LC-MS as the method of choice. LC-MS fingerprint data are typically processed as follows prior to chemometrics analysis: 1) determining molecular features, then 2) retention time alignment, 3) bucketing, 4) filtering, scaling, normalization, and finally 5) data analysis (Indrayanto, 2018b). Automatic time alignment of R_t_-m/z pairs for certain time intervals and mass ranges (e.g. 100–1,000 Da) can be performed using software supplied with the LC-HRMS instrument. Data can be grouped automatically into buckets with R_t_-m/z pairs (*x* minutes-*a* Da) with a mass tolerance of *b* Da (Ratih et al., 2019). Detailed methods for processing and bucketing the raw LC-MS data have been discussed in a previous publication (Thiele et al.). Recently, a new algorithm based on sub-window factor analysis (SFA)-HRMS for peak alignment of LC-HR-MS data was proposed (Zeng et al., 2021). Initially, region of interest (ROI) searching and fuzzy matching are combined to transform the raw data sets into equidistant matrices effectively. Protocols for data pre-treatment, processing, and validation for chromatographic fingerprinting using LC-MS and GC-MS have been described in detail in previous publications (Want et al., 2010; Blaise et al., 2021).

Third-order chromatographic fingerprints can be generated by using LC-MS/MS, GC-MS/MS (R_t_, m/z_1_, m/z_2_), or 2D-LC/GC-DAD/MS (data: R_t1_, R_t2_, wavelength, or m/z) (Li et al., 2020b). Algorithms and workflows for effective chromatographic fingerprinting are available within the majority of commercial software packages dedicated to GCxGC and other comprehensive two-dimensional chromatography platforms (Stilo et al., 2020). The theory, experiments, and various chemometrics processing methods of multi-way chromatographic methods (e.g., 2D-LC- DAD/MS, 2D-GC-MS, GC-MS/MS, three-dimensional (3D)-GC-MS) have been previously discussed (Escandar and Olivieri, 2019). The application of multivariate curve resolution-alternating least-squares (MCR-ALS) and parallel factor analysis (PARAFAC) for multi-way chromatographic methods have also been recently reviewed (Anzardi et al., 2021).

Qualitative and quantitative chromatographic fingerprinting methods can be applied as QC tools in all stages of herbal drugs production i.e., incoming materials, in-process control, finished products, and stored samples (Indrayanto, 2018a). As described in Section 2, the official identification method for herbs (incoming materials) is comparing the HPTLC fingerprint of the samples to the authentic standards or BRMs visually. Due to some limitations in visual observation, evaluation of the chromatographic fingerprints by calculating similarity values (R, c) and/or multivariate analysis (PLS-DA, SIMCA, LDA, etc.) is more recommended. The application of second-order chromatographic fingerprinting (GC-MS, LC-MS) will yield more accurate results for herb identification.

To have the best consistency in each production batch, the composition of herbs/extracts should be evaluated qualitatively and quantitatively for every stage of the production process (Heinrich et al., 2020; Wei et al., 2020). The quality assessment of herbal drug preparations is typically not easy due to the complex nature of the metabolite contents and their possible variation. It is well known that many external factors can affect the metabolite contents qualitative and quantitatively. Extraction methods and material processing could also affect the stability of the metabolites. The availability of BRMs and stable standardized extracts are crucial for the quality assessment of each stage of herbal drug production. Some recent publications on the application of chromatographic fingerprinting for QC assessment of herbal drugs during their production are discussed below.

Yan et al. proposed the applications of macro qualitative similarity (S_m_) and macro quantitative similarity (P_m_) for evaluating the compositions (qualitative, semi-quantitative) of the herbal drug preparations during production using HPLC-UV fingerprint (Yan et al., 2021). Reference fingerprint (RFP) of the standard preparation (SP) was used. Establishing an SP for an herbal drug requires multi-source raw materials or preparations; generally, not less than 15 batches of samples for a single raw material and 100 batches of samples for a herbal preparation. A qualified product requires its S_m_ ≥ 0.90, 80% ≤ P_m_ ≤ 120%, and qualified content of markers. The standard value of RFP should be S_m_ ≥ 0.95 and P_m_ ≈ 100. Yao et al. applied a multi evaluation method for a non-prescription herbal drug mixture using ultra-performance liquid chromatography (UPLC) fingerprinting at 254 nm, compound identification via UPLC-triple time of flight (Triple-TOF)-MS analysis, and quantitative determination of the compounds via UPLC-UV-254 nm (Yao et al., 2019). Although similarity levels of the fingerprints were 0.935–0.984, PCA using 7 detected compounds showed 2 clusters. These results showed that the application of first-order chromatographic fingerprinting has limitations. Ji et al. compared the profiles of compounds in traditional Chinese medicine (TCM) tablets and their herbal components using 2D-LC-quadrupole-TOF (q-TOF) MS (Ji et al., 2018). Fifty-four compounds were identified from the total 465 peaks found in the tablet samples. Twelve out of 465 peaks could not be found in the chromatograms of the herbal components, suggesting further investigation on the compatibility of the herbal components. Differences in LC-q-TOF MS profiles of raw materials and processed Chinese herb preparation have also been reported (Li et al., 2021). These differences affected the disposition of metabolites in an *in-vivo* study using rats. Another group (Li H. et al., 2020) applied multiple fingerprint profiling of polysaccharides in Chinese traditional drugs using FTIR, HPLC-UV, and size exclusion chromatography (SEC)-RID for quality determination. Twelve batches of these herbal drugs showed a high degree of similarity. Unfortunately, the authors did not discuss the advantages of using fingerprints from polysaccharides in comparison with those from the whole metabolites. Zhao et al. studied the LC-MS profiles and anti-proliferative effects of 22 commercial gingers (Zhao et al., 2020). These data were then evaluated using PCA. The study demonstrated that variation in the fingerprint profiles that results from differences in chemical compositions could have a significant impact on the efficacy and bioactivity of the ginger extracts.

The discussions above show that the quality assessment of herbs and/or plant extracts for research and QC purposes can be only well performed using chromatographic fingerprinting if the authentic BRM and/or standardized extract are available and possess desired pharmacological activities. Identical chromatographic fingerprints mean the sample is phyto equivalent to its standard (Sahoo et al., 2010). It would also be useful if the regulatory agency in each country could produce official BRMs for all herbs. Due to many environmental factors that can affect the metabolite contents, the application of official BRMs from other countries is not recommended (Bensoussan et al., 2015; Indrayanto, 2018b). For QC in the production lines, pharmaceutical industries should develop a standardized mixture of herbs and/or extracts with exact compositions. Without these standardized mixtures, it is impossible to have herbal drugs with consistent efficacy. Further research should be conducted to determine the quality markers of each herb/product to avoid the need for performing chemical profiling at the QC laboratories in pharmaceutical industries.

### 4.3 Electrophoretic Fingerprinting

Fingerprinting using electrophoresis presents great advantages for the analysis of complex herbal medicines due to its separation capability. Electrophoretic techniques have been used for decades to assist both molecular and chemical fingerprinting. Charged molecules such as nucleic acids and metabolites can be well separated using these techniques to obtain fingerprints (Shen et al., 2019; Gong et al., 2021). Gel electrophoresis using agarose or polyacrylamide gel is often employed to quickly confirm the presence of certain amplification products or perform sequencing in DNA barcoding and fingerprinting (Shen et al., 2019; Hadipour et al., 2020). Capillary electrophoresis (CE) has also been extensively explored in chemical fingerprinting for the standardization and quality control of herbal medicines (Johnson and Lunte, 2016; Gong et al., 2021).

CE has been used for fingerprint analyses of various raw materials and products containing medicinal plants such as *Ginkgo biloba* (Johnson and Lunte, 2016), *Smilax glabra* Roxb. (Zhang and Cheung, 2011), *Carthamus tinctorius* L.(Sun et al., 2003), *Scutellaria baicalensis* (Yu et al., 2007; Wang Y. et al., 2014), *Dendrobium candidum* (Zha et al., 2009), *Glycyrrhiza uralensis* (Ma et al., 2017; Gong et al., 2021), etc. Gong et al. recently reported a quality evaluation of Compound Liquorice Tablets (a Western-Chinese herbal preparation containing liquorice extract, powdered poppy, camphor, star anise oil, and sodium benzoate) using CE with UV detection (Gong et al., 2021). Tablets were ground, dissolved in 0.5% phosphoric acid-acidified methanol-water (80:20 v/v) mixture, filtered, and subjected to CE separation using a fused silica capillary at 15 kV separation voltage and 25 mM sodium tetraborate: acetonitrile (5:1) background electrolyte.

The composition of background electrolyte (BGE) plays a pivotal role in fingerprint analysis using CE. The electrophoretic mobility of the analytes is dictated by the hydrated radius and overall charge of the molecules. Therefore, buffered solution at a certain pH is often used as a component in BGE to protonate or deprotonate neutral molecules. For example, the pH of the BGE may be set in the range of 8.7–10.0 using borate buffer in the analysis of HMs containing flavonoids and phenolic compounds so that the compounds will carry at least one negative charge (Chen et al., 2011; Johnson and Lunte, 2016; Roblová et al., 2016). In addition to providing buffering capacity in this pH range, borate can form complexes with the phenolic compounds and increase their overall sizes and effective charges (Johnson and Lunte, 2016). For example, Johnson and Lunte used 25 mM ammonium biborate in 10% methanol (pH 9.3) to perform CE separation of 14 flavones, flavanones, and flavonols with similar structures and several representative glycosides from plants (Johnson and Lunte, 2016). Acidic BGE such as a mixture of methanol-acetonitrile (85:15 v/v) containing acetic acid 0.5% and ammonium acetate 90 mM was reported for the analysis of alkaloids from Amaryllidaceae (Gotti et al., 2006). Under this condition, the alkaloids (i.e., galanthamine and haemanthamine) are protonated and can be resolved from one another using CE. In another experiment on CE fingerprinting of alkaloids from *Sophora flavescens* (Hou et al., 2019), coordination additives were found to play a larger role in CE separation than pH condition does. The additives possibly assisted the boron anion complexation with alkaloids, enhancing the differences in mobility among analytes.

Similar to chromatographic fingerprints, qualitative and quantitative analysis using CE fingerprints can be performed in multiple ways. One of the reported approaches is classification into several quality grades based on the quantitative fingerprinting method (QFM). In this method, the similarity of a sample to reference material is evaluated based on several criteria and then the sample is assigned to one of the 8 grades (i.e., 1 = best, 2 = better, 3 = good, 4 = fine, 5 = moderate, 6 = common, 7 = defective, and 8 = inferior) based on the similarity level (Liu et al., 2015; Gong et al., 2021). Samples that fall into grades 1–5 are considered qualified. QFM typically uses 3 criteria to determine the quality grade of the sample: 1) qualitative similarity, which represents the similarity in the number and distribution of fingerprint peaks between the sample and reference, 2) quantitative similarity, which reflects the similarity in the overall content of the fingerprints, and 3) fingerprint variation coefficient, which represents the qualitative variation/dissimilarity of the fingerprints (Liu et al., 2015). Based on how these 3 criteria are calculated, several QFMs have been reported such as the simple quantified ratio fingerprint method (Liu et al., 2015), limited ratio quantified fingerprint method (Chen et al., 2018), equal weight ratio quantitative fingerprint method (Gong et al., 2021), linear quantitative profiling method (Hou et al., 2019), averagely linear quantified fingerprint method (Zhang et al., 2019), systematic quantified fingerprint method (Lan et al., 2019), and average method of systematic quantified fingerprint method (Wang et al., 2021b).

The relationship between the CE fingerprints and other properties such as biological activities can also be further investigated using PLSR (Chen et al., 2018; Hou et al., 2019). Other chemometric methods for classification such as PCA and HCA have also been explored on CE fingerprints (Iino et al., 2012; Roblová et al., 2016; Hou et al., 2019). Validation of CE methods typically follows the same protocols and criteria as those of chromatographic methods. CE methods are validated by evaluating their linearity, limit of detection and quantification, precision, and accuracy, whereas the chemometric calibration models are evaluated based on the R^2^ and RMSE values (Hou et al., 2019).

CE fingerprinting methods offer advantages for the standardization and quality control of herbal medicine such as low reagent consumption, relatively high analysis speed, and improved separation efficiency. However, since analytes are separated based on their electromigration, the separation of neutral metabolites such as terpenes in CE may be challenging. This could be a problem if neutral metabolites are major constituents and play a significant role in HM differentiation/classification. Adding another separation dimension by performing micellar electrokinetic chromatography (MEKC) and/or using an MS detector could potentially solve the issue. MEKC improves the separation of neutral compounds by combining the electrophoretic-electroosmotic mobility of the analytes and their partitioning between BGE and surfactant micelles (Liu et al., 2015). MS can help separate unresolved peaks into their constituents, provided that none of the constituents experiences severe ionization suppression under the CE-MS condition. Alternatively, chromatographic separation can be opted for.

### 4.4 Direct MS Fingerprinting

Not only is MS a powerful detector in hyphenated techniques such as HPLC-MS and CE-MS, but it has also been utilized to perform direct fingerprint analyses for herbal standardization and quality control. Direct MS has been used to collect fingerprints of herbal materials from a variety of plants such as *Allium sativum* (Pereira et al., 2021), *Fritillaria sp.* (Xin et al., 2014; Wang et al., 2017), *Panax quinquefolium* L. (Chan et al., 2011), *Origanum sp.* (Massaro et al., 2021), *Gastrodiae elata* (Wong et al., 2016), *Cynanchum sp*. (Jun et al., 2016), etc. Ambient MS in which analytes are ionized at ambient pressure is often employed (Xin et al., 2014; Massaro et al., 2021; Pereira et al., 2021), although inductively coupled plasma (ICP) MS for multi-element fingerprinting (Zhao et al., 2017) and matrix-assisted laser desorption/ionization (MALDI) MS (Lai et al., 2012) have also been reported. Ambient ionization techniques applicable to herbal materials and products include desorption electrospray ionization (DESI) (Talaty et al., 2005; Freitas et al., 2019), ESI on solid substrates (Deng and Yang, 2013; Hu et al., 2014; Wang H. et al., 2014; Xin et al., 2014; Jun et al., 2016), tissue ESI and related techniques (Chan et al., 2011; Liu et al., 2011; Hu et al., 2013), extractive ESI (Lee et al., 2021; Zhang et al., 2022), direct analysis in real-time (DART) (Kumar et al., 2015; Wang et al., 2021a), and desorption atmospheric pressure chemical ionization (DAPCI) (Pi et al., 2011).

In DESI, analytes in herbal samples are ionized/desorbed by impinging the surface of the samples using charged solvent droplets. This technique allows for the analysis of samples without any pretreatment. DESI also enables surface imaging for mapping the spatial distribution of secondary metabolites within the sample specimen (Freitas et al., 2019). DESI has been applied to raw herbal materials such as leaves, stems, roots, and flowers as well as dosage forms such as tablets and capsules (Yang and Deng, 2016). DESI on powdered samples may be challenging because the powder could spatter after being hit by the solvent droplets, hindering analyte desorption and/or ion transfer to the mass analyzer. To overcome this problem, powdered samples can be compressed into thin tablets or dissolved in a volatile solvent and applied as a thin film on a solid surface prior to MS analysis (Crawford et al., 2017).

Similar strategies are used in solid substrate-based ESI techniques in which samples are placed onto/into solid supports such as triangular paper (Deng and Yang, 2013), wooden toothpicks (Xin et al., 2014; Pereira et al., 2021), aluminum foil (Hu et al., 2014), and pipette tips (Wang H. et al., 2014). Paper and wooden toothpicks are porous materials that can hold samples. Paper-spray ionization is typically applied to liquid samples (Deng and Yang, 2013), whereas the wooden tip ESI can be applied to both liquid and solid samples (Xin et al., 2014; Pereira et al., 2021). The toothpick can be directly dipped into liquid samples or pre-wetted with a suitable solvent followed by dipping into solid samples. A larger quantity of samples such as bulk materials can be loaded into a folded aluminum foil or pipette tip (Wang H. et al., 2014; Hu et al., 2014). The pointed side/tip of the triangular paper, toothpick, folded foil, or pipette tip is then placed toward the MS inlet while applying a high voltage to the sample/support for ionizing the analytes. Alternatively, the high voltage can be applied to the MS inlet while the sample is grounded (Hu et al., 2013). This technique can preserve the sample and therefore, is suitable for real-time monitoring of secondary metabolites in unharvested medicinal crops. Pipette tips loaded with powdered samples can also be connected to a solvent-filled syringe to perform simultaneous extraction and spray ionization (Wang H. et al., 2014).

DART produces analytical results similar to those of DESI. The difference between the techniques lies in the medium used to ionize the samples. In DESI, the sample surface is exposed to an electrospray plume, while an ionizing noble gas stream (i.e., metastable He) is used in DART (Gross, 2006). Using DART, botanical samples can be directly analyzed without any sample preparation (Kumar et al., 2015; Wang et al., 2021a). Ionizing gas is also used in DAPCI to desorb/ionize analytes from the plant samples (Pi et al., 2011). A high voltage is applied to produce a corona discharge that ionizes the carrier gas (e.g., He, N_2_, or Ar). This gas is then pneumatically directed to the sample surface.

After obtaining mass spectra fingerprints, multivariate data analysis can then be applied to the datasets to extract the information of interest. Exploratory data analysis via PCA is often done to reveal certain grouping or clustering of medicinal samples based on the characteristics of MS fingerprints (Pi et al., 2011; Xin et al., 2014; Pereira et al., 2021). The exploratory analysis only tells whether known samples can be differentiated from one another based on the score plot. To predict which class or cluster an unknown sample belongs to, prediction models using supervised classification methods can be implemented. For example, Zhang et al. recently performed PCA and OPLS-DA on 90 *P. notoginseng* samples grown under different conditions (Zhang et al., 2022). Results from the PCA and OPLSDA are in agreement in which there were 12–14 MS peaks that mainly contributed to the differentiation. These peaks, included sucrose, fructose, several ginsenosides (Rg1, Rf, Rb1, Noto-R1, malonyl-Rb1, malonyl-Rg1, malonyl-Rf, Rd, and Re), linoleic acid, palmitic acid, and malic acid, can be used as key indicators to discriminate samples of different origins, commercial specifications, and growing conditions. The parameters R^2^Y and Q^2^ of the OPLS-DA model were 0.939 and 0.875, respectively, showing good predictive ability. Prediction models based on PLS-DA and MS fingerprints have also been built for other plant materials, providing up to 97% accuracy in the prediction rate (Xin et al., 2014; Pereira et al., 2021).

Massaro et al. validated a DART-MS fingerprinting method for oregano authentication by SVM (Massaro et al., 2021). They first conducted exploratory PLS-DA on 4 independent data sets (i.e. spectra collected with two different extraction solvents and two ion modes in MS). The most discriminated variables were selected from each data set and then merged using a mid-level data fusion approach. These variables were used to build an SVM classifier which was then validated by Monte Carlo cross-validation and against an independent set of oregano samples (external validation). A 90% prediction accuracy was reported with specificity and sensitivity of 92% and 95%, respectively. Incorrectly classified samples included samples containing adulterants not used in building the prediction models, showing that the classification ability of the model largely depends on the calibration data sets. Another authentication study based on direct MS fingerprints was carried out by Wang et al. using PLSR (Wang et al., 2017). Pure *Fritillaria unibactreata* was mixed with adulterants at 0%–100% w/w concentrations to develop the prediction model. Linearity and reproducibility of the method were assessed using QC samples containing pure herbs at several concentrations in five replicates. The R^2^ and RMSEP of the prediction model were 0.9072 and 0.1004, respectively. The model especially suffered from a lack of accuracy when used to analyze samples at low concentrations of target herbs (<10%). Although this was not the best system, the model can still be useful for the rapid screening of adulterated samples. Better results were obtained from PLSR models constructed using hyphenated techniques such as UPLC-q-TOF/MS and UPLC-triple quadrupole (TQ)/MS fingerprints (Wang et al., 2017), suggesting that interference from matrix components may be prominent in direct MS.

The main advantage of performing direct MS over hyphenated methods is the speed of analysis and high sampling throughput. In addition, several direct MS techniques allow minimum destruction to the sample which is desirable in real-time monitoring/fingerprinting of metabolites in living organisms. This benefit can be exploited for rapid monitoring of metabolite profiles in unharvested plants to determine the optimum growing conditions or harvesting age. However, direct MS may suffer from matrix effects which could yield inaccurate identification and/or quantification. Increasing the number of sampling spots and/or using extractive ESI to selectively take the metabolites out of the plant matrix could potentially mitigate the issue. Nevertheless, the method must be thoroughly validated to ensure the reliability of the results.

### 4.5 DNA Barcoding and Fingerprinting

DNA analysis has been used in herbal drug research to perform 1) authentication of medicinal plants, 2) detection of adulteration or substitution with other closely related species, 3) breeding of medicinal plants, and 4) quality control and standardization of medicinal plant materials. In the context of plant authentication, DNA analysis is mostly used to differentiate among plant species, not individual plants within the species. This technique is often referred to as DNA barcoding. For example, Shen and coworkers reported authentication of *Drynaria rosii*, a traditional Chinese herb using DNA barcodes (Shen et al., 2019). To develop the barcodes, genomic DNA of *D. rosii* and other 6 closely related species (*D. sinica, D. bonii, D. delavayi, D. quercifolia, D. propinqua*, and *Pseudodrynaria coronans*) were polymerase chain reaction (PCR)-amplified, sequenced using the Sanger method, and aligned to generate a phylogenetic tree. The tree provided an evident clustering in which plant samples from the same species were clustered into one clade, showing its potential for differentiating *D. rosii* from adulterants. However, complications may arise if the tested sample is a mixture of herbs. The presence of sequences from multiple species may affect the placement of the sample within the clusters, hampering definite identification. Chosen markers for the authentication of articles of botanical origin must be specific enough to identify target and adulterant species in the samples, but also universal enough to prevent false-negative from closely related species (USP44-NF39, 2021c).

Various DNA barcodes have been made available online to assist authentication of herbal materials. Several barcoding databases that can be used include the Barcode of Life Data System (http://www.boldsystems.org), IdIt-ITS2 (http://its2-plantidit.dnsalias.org), PTIGS-IdIt (http://psba-trnh-plantidit.dnsalias.org), and Medicinal Materials DNA Barcode Database (http://www.cuhk.edu.hk/icm/mmdbd.htm) (Chen et al., 2014). The Consortium for the Barcode for Life (CBOL) proposed 7 plastid DNA regions for plant DNA barcoding including *atp*F*-atp*H*, rbc*L*, rpo*B*, rpo*C1*, mat*K*, psb*K*-psb*I*,* and *trn*H*-psb*A (CBOL-Plant-Working-Group et al., 2009)*.* Many other barcodes have been reported in the literature and the choice of barcodes has been discussed in several review articles (Hollingsworth et al., 2011; Li et al., 2015; Zhokhova et al., 2019). Typically, these barcodes were evaluated based on their discriminating abilities. Combinations of barcodes from different loci are typically proposed to improve efficiency in plant species discrimination. CBOL recommended the 2-locus combination of *rbc*L and *mat*K for its ability to successfully discriminate species in 72% of cases and discriminate congeneric species in 100% of cases (CBOL-Plant-Working-Group et al., 2009). Another study revealed barcodes derived from the ITS2 region provided better discrimination efficiency for medicinal plants than the commonly used *rbc*L gene, with a discrimination efficiency of more than 90% at the species level (Chen et al., 2010; Zhang et al., 2015). ITS2 barcodes are relatively short (∼200 bp) which are favorable for identification and quality control in herbal preparations, since plant DNA in these samples is often significantly degraded to <500 bp fragments (Zhokhova et al., 2019). A combination of ITS2 and *psb*A-*trn*H barcodes are available for most herbal plants listed in the Chinese, Japanese, Korean, Indian, United States, and European Pharmacopoeias (Chen et al., 2014). DNA barcodes for common medicinal plants in the tropics have also been reported (Tnah et al., 2019).

While these barcodes can discriminate even closely related species, co-amplification of the barcoding sequences in herbal preparations containing multiple plant species or excipients may negatively impact the DNA decoding. Also, the addition of excipients in large amounts may cause the barcode primers to preferentially amplify DNA from the excipients (Zhokhova et al., 2019). To overcome these problems, digital PCR or next-generation sequencing (NGS) can be integrated into the barcoding protocol. In digital PCR, DNA samples are diluted in a suitable buffer at several dilution ratios such that the final DNA concentration for the PCR template is approximately 1 molecule per μL (Morley, 2014; Zhokhova et al., 2019). Using this approach, a mixture of DNA from different plant sources or materials can be deconvoluted to improve the chance of low-abundant DNA molecules being amplified and detected. This method has been used for authentication of *Ginkgo biloba* in herbal dietary supplements (Little, 2014). Droplet digital PCR was reported by Yu et al. for qualitative and quantitative analysis of *Panax notoginseng* powder samples mixed with several adulterants (Yu et al., 2021). NGS allows for independent amplification of individual DNA sequences within the mixture and excludes any overlapping DNA sequences, enabling more accurate DNA decoding in multi-component herbal preparations. This approach has been applied to the authentication of various herbal supplements containing *Echinacea purpurea*, *Valeriana officinalis*, *Ginkgo biloba*, *Hypericum perforatum*, and *Trigonella foenum-graecum* (Ivanova et al., 2016)*.* The applications of NGS for the identification and authentication of herbal products have been discussed in several review articles (Haynes et al., 2019; Lo and Shaw, 2019).

Although significant progress has been made, DNA barcoding in herbal preparations containing multiple components remains challenging. Method validation should always be performed to evaluate the reliability of the results. AOAC International has published guidelines for the validation of botanical identification methods (qualitative) and quantitative chemical methods for dietary supplements and botanicals (AOAC, 2019). However, translating these guidelines to DNA-based methods may not be trivial. DNA barcoding methods are often validated using raw plant materials and therefore become less appropriate for detecting adulterants in finished products. When developing a DNA barcoding method for detecting adulteration, it is also important to determine the limit of detection for each adulterant by creating mixtures of the target species with known amounts of possible adulterants.

DNA fingerprinting is a method for simultaneously detecting mini- or microsatellites (i.e., short sequences of repetitive DNA that show greater variations among individuals) to create a unique pattern for identification. DNA fingerprinting techniques are especially useful for plant genotyping and controlling the quality of medicinal crops (Zhokhova et al., 2019). Because variations in the plant genetic materials may affect the phenotypes, including the production of secondary metabolites, identification of plant varieties with desired traits could assist in preparing more standardized plant materials with similar characteristics. In the early days of DNA fingerprinting, restriction fragment length polymorphism (RFLP) in conjunction with Southern blot hybridization became the main technique for profiling plants’ DNA (Dallas, 1988; Antonius and Nybom, 1994). This technique, however, is time-consuming and requires multiple species-specific probes, limiting its applications mainly to economically important plants.

PCR-based DNA fingerprinting techniques have been reported in which single oligonucleotide primers with random sequences were used to produce PCR fragments from multiple loci in the genomic DNA (Welsh and McClelland, 1990; Caetano-Anolles, 1991). One of the techniques that became popular was random amplified polymorphic DNA (RAPD), in which single primers were used to amplify nonspecific sites of the DNA (Chang et al., 2017; Rafalski et al., 2020). For example, RAPD markers were used to assess the genetic diversity of *Curcuma comosa* Roxb and other *Curcuma* sp. collected from different regions in Thailand. Fifteen RAPD primers were used to amplify the genomic DNA from 30 plant samples (Boonsrangsom, 2020). The RAPD profiles were then used to classify the samples into two major clusters: Cluster I which consists of *C. comosa* Roxb samples from different regions and Cluster II which consists of other *Curcuma* sp. Cluster I was divided into 6 sub-clusters which may be useful for conservation and breeding programs by further analyzing the metabolite profiles of the samples and the correlation between the genetic and metabolite profiles.

Amplified fragment length polymorphism (AFLP) and inter-simple sequence repeat (ISSR) techniques are also common (Paun and Schönswetter, 2012; Hassan et al., 2020; Junior et al., 2020; Leipold et al., 2020). AFLP uses selective amplification of digested DNA fragments to generate unique DNA fingerprints, while ISSRs are DNA fragments (100–3,000 bp) located between two adjacent, oppositely oriented microsatellite regions. Hadipour et al. investigated the genetic variation of wild *Papaver bracteatum* L. from 9 different populations in Iran using AFLP and ISSR markers (Hadipour et al., 2020). The genetic diversities were 52% and 48% among different populations; 38% and 41% within the populations for ISSR and AFLP, respectively. AFLP and ISSR also similarly grouped the samples into 3 major groups and 1 minor group, which correlated well with the geographical distribution of the samples.

While DNA barcoding and fingerprinting are powerful tools for medicinal plant genotyping and authentication, there are several limitations associated with these methods. The successful application of DNA barcoding/fingerprinting relies on the quality of DNA, primer affinity, amplification, and amplicon sequencing. Intact plant DNA can typically be extracted from fresh or dried materials using standard DNA extraction methods (Shen et al., 2019; Hadipour et al., 2020). However, significant degradation of DNA can occur during the manufacturing process of herbal products (de Boer et al., 2015). Thus, DNA-based methods are more appropriate for the initial stage of raw material preparation and standardization rather than for the quality control of highly processed herbal preparations. These methods will also not be able to determine from which plant part the materials come and cannot be used in the case of adulteration with different parts of the same plant species. Plant DNA profiles may not be well correlated with the secondary metabolite contents as genomic DNA remains unaffected by seasonal variations, whereas metabolite production can vary between seasons (Ahmed et al., 2019). In addition, the removal of certain metabolites during extraction or other processes will not be reflected on the DNA profiles. Therefore, DNA barcoding/fingerprinting cannot be single-handedly used to predict the efficacy of raw materials or finished products and must be used to complement the chemical analysis and macroscopic/microscopic evaluation of the herbal specimen/samples.

## 5 Conclusion, Challenges, and Future Perspectives

Due to the complex nature of herbal drug preparations, the method of standardizations for individual herbs that are described in the herbs’ monographs in the pharmacopeia cannot be applied directly as a QC tool for all stages in the manufacturing processes, except for the quality assessment for the incoming herbs/extracts. The visual evaluation based on the HPTLC method described by the Pharmacopoeias and official guidelines should be completed using similarity- and/or chemometrics-methods. Chemical profiling or fingerprinting is the method of choice for performing quality control if the quality markers are not yet specified for each of the herbs.

Using fingerprinting methods, various tasks in the research and development of herbal drugs can be performed. Chemical fingerprints can be used to evaluate the quality of raw materials, extracts, and finished products. With the help of chemometrics, information can be extracted from the fingerprints to find similarities/differences which are useful to group samples based on certain characteristics (e.g., authentic vs adulterated samples, samples from different geographical origins, etc.) and establish correlations between the chemical profiles and biological/pharmacological activity of interest. Relationships between chemical profiles/fingerprints and biological/pharmacological activities such as antioxidant, antibacterial, antihypertensive, anti-inflammatory, and antitumor have been successfully established in multiple plant materials and HM preparations (Zhang et al., 2018). Results obtained from these studies have also led to the discovery of quality markers that can be used for future QC applications.

Chemical fingerprints can be obtained using various separation-based (e.g., LC, TLC, and CE) or nonseparation-based (e.g., FTIR and NMR spectroscopy) techniques. Due to their ability to rapidly generate chemical fingerprints, chemical profiling using direct spectroscopy and MS methods may offer more benefits over chromatographic methods. The application of FTIR is preferred due to its relatively lower operation cost. Using single measurements, spectroscopic/spectrometric methods can generate a large number of spectral data which can be characterized by wavenumbers, intensities, chemical shifts, or mass-per-charge. By optimizing spectral treatments (pre-processing), selection of fingerprint regions, and the use of appropriate chemometric techniques, these methods can be applied for the standardization and quality control of HMs. Assessment of both chemical dan DNA fingerprints would provide a more comprehensive outlook on the authenticity and overall quality of the HMs, and thus are recommended to be used in conjunction when appropriate. The combined assessment would be especially useful to determine plant genotypes that result in desirable phenotypes such as high contents of certain bioactive metabolites. In addition, collaborative studies through proficiency testing are required to get comparable results for these fingerprinting methods and to finally propose them as standard methods in the future.

To have reliable results for the quality assessment using chemical profiles, the availability of authentic botanical reference materials and stable standardized extracts is crucial. Although the current pharmacopeias have described the physicochemical specifications of each raw plant material, the active component(s) and associated pharmacology activities are not specified. In addition, these individual specifications cannot be directly applied to determine the specification of HM containing multiple plant materials. Therefore, pharmaceutical industries should prepare and provide stable standardized extracts that have certain pharmacological applications for their QC. It would be practical if specifications of commonly used HM preparations including their active components and desired therapeutic applications are provided in the herbal or general compendia in the near future.

Many studies reported to date are still limited to plant materials, dried mixtures of plant materials, or extracts without reporting their exact chemical compositions, making comparison among results found in the literature and replicating the experiments difficult. To prevent these problems, the chemical compositions of extracts or HM preparations should be accurately determined, and all methods used for the chemical, biological, and/or pharmacological testing (*in-vitro*, *in-vivo*, *ex-vivo*) should be fully validated according to the newest guidelines, prior to routine application (Indrayanto, 2022). Finally, appropriate clinical trials should be conducted before HMs can be prescribed and used in clinical settings. The exact chemical compositions of the HMs and their stability must be determined to assure similar efficacy from batch to batch. Knowing the exact composition of the HM may also assist in determining incompatibilities between the active components and excipients, and possible unwanted interactions between the HM components and other drugs or food. The pharmacokinetic parameters of the HMs must be evaluated to ensure effective and safe use of HMs.
